# Regulatory framework for polymer-based nanotherapeutics in clinical translation

**DOI:** 10.3389/fbioe.2026.1735885

**Published:** 2026-01-21

**Authors:** Federico Stucchi, Min Li, Giuseppe Castellano, Francesco Cellesi

**Affiliations:** 1 Department of Chemistry, Materials and Chemical Engineering “Giulio Natta”, Politecnico di Milano, Milan, Italy; 2 Renal Research Laboratory, Fondazione IRCCS Ca' Granda Ospedale Maggiore Policlinico, Milan, Italy; 3 Unit of Nephrology, Dialysis and Renal Transplant, Fondazione IRCCS Ca' Granda Ospedale Maggiore Policlinico, Milan, Italy; 4 Department of Clinical Sciences and Community Health, University of Milan, Milan, Italy

**Keywords:** clinical translation, critical quality attributes, nanomedicine policy, polymer conjugates, polymeric nanoparticles, quality by bench-to-bedside design, regulatory framework

## Abstract

Polymer-based nanotherapeutics (PNs) have emerged as versatile drug delivery platforms offering controlled release, targeted delivery, and improved pharmacokinetics across therapeutic areas. However, their structural complexity and nanoscale behavior pose significant regulatory challenges. Current frameworks still evaluate PNs under conventional pharmaceutical and biological regulations, overlooking nanospecific properties and leading to case-by-case assessments. This review examines critical quality attributes, analytical requirements, and gaps in standardization that hinder clinical translation. Key issues include inadequate characterization of the physicochemical properties, scalability of manufacturing, and limited predictability of *in vivo* performance. Highlighting the mismatch between nanoscale properties and existing regulatory expectations, this review underscores the need for a harmonized, science-driven framework to ensure safety, efficacy, and global acceptance of PNs.

## 1 Introduction

Polymer-based nanotherapeutics (PNs), such as polymeric nanoparticles, nanocarriers and polymer conjugates, have emerged as one of the most versatile platforms in nanomedicine, offering controlled drug release, targeted delivery, and improved pharmacokinetics for a wide range of therapeutic agents (Kamaly et al., 2012; Beach et al., 2024). Their tunable composition, surface functionality, and biodegradability make them particularly attractive for oncology, infectious diseases, and gene therapy applications (Karlsson et al., 2018; Cano et al., 2020; Li et al., 2024). However, their structural complexity and nanoscale behavior challenge traditional pharmaceutical evaluation paradigms. Unlike conventional small molecules, PNs exhibit properties, such as size-dependent biodistribution, dynamic surface interactions, and potential immunogenicity, that are highly sensitive to manufacturing conditions and formulation design (Elsabahy and Wooley, 2012; Beach et al., 2024).

Regulatory authorities, including the European Medicines Agency (EMA) and U.S. Food and Drug Administration (FDA), currently assess nanotechnology-based medicines under existing pharmaceutical and biological product frameworks (EMA/HMA, 2025). However, this regulatory practice does not fully account for nanoscale-dependent behaviors, such as size-driven biodistribution, dynamic surface interactions, and high sensitivity to manufacturing variations, that fundamentally distinguish PNs from traditional medicines. As a result, the evaluation of PNs often relies on case-by-case judgments, reflecting the absence of dedicated nanomedicine-specific standards and contributing to uncertainty and inconsistency in regulatory expectations. Clarifying these limitations is essential to understanding why existing frameworks remain insufficient for ensuring adequate and scientifically robust assessment of PNs.

This review examines the current regulatory landscape governing PNs in clinical translation, highlighting existing frameworks, methodological gaps, and global harmonization efforts. By analyzing critical quality attributes, analytical requirements, and methodological gaps, and by discussing emerging approaches such as Safe-by-Design and artificial intelligence (AI) tools, the review underscores the need for a more tailored, science-driven regulatory paradigm to support predictable and consistent clinical development of PNs.

## 2 Scientific background

PNs have been explored as versatile carriers in nanomedicine and drug delivery, offering precise control over size, shape, surface properties, and chemical functionality (Kamaly et al., 2016; Gagliardi et al., 2021; Mitchell et al., 2021). Their modular design allows encapsulation of small molecules, proteins, and nucleic acids while enabling controlled release at targeted sites (Mitchell et al., 2021; Machtakova et al., 2022; Rodriguez-Cruz et al., 2025). Clinical successes of PNs such as protein-polymer conjugates (Moncalvo et al., 2020), drug-loaded polymeric micelles (Park et al., 2017), synthetic polypetides (Aharoni et al., 2024) highlight their therapeutic promise. However, widespread clinical adoption is limited due to biological barriers, patient variability, and challenges in translating preclinical findings. Ongoing research focuses on rational design of polymers and nanostructures to overcome clearance mechanisms, enhance targeting, and achieve stimulus-responsive delivery (Luo et al., 2023; Beach et al., 2024).

### 2.1 Polymeric materials

PNs are fabricated from diverse polymers with distinct chemical and biological properties. Natural-derived polymers include polypetides (Yuan et al., 2025) and polysaccharides (chitosan, hyaluronic acid) (Serrano-Sevilla et al., 2019), which offer biocompatibility and intrinsic bioactivity. Glycopolymers and phosphate-based polymers mimic biological interfaces and support biodegradability (Stenzel, 2022). Synthetic polymers dominate due to tunable structures: polyesters such as poly (lactic acid) (PLA), poly (lactic-co-glycolic acid) (PLGA) and poly (ε-caprolactone) (PCL) are FDA and EMA approved for controlled release (Schoubben et al., 2019). Cationic polymers such as polyethyleneimine (PEI) enable nucleic acid delivery, though PEI is limited by cytotoxicity (Pereira et al., 2022; Ismail and Chou, 2025). Poly (ethylene glycol) (PEG) is widely employed to prolong circulation (“stealth” effect), though alternatives such as poly (2-oxazoline) and zwitterionic polymers are increasingly explored due to anti-PEG immune responses (He et al., 2024; Golba et al., 2025). Stimuli-responsive polymers (pH-, redox-, enzyme-, or temperature-sensitive) allow site-specific release in tumor or infection microenvironments (Chang et al., 2021). Collectively, this chemical toolbox provides a foundation for tailoring nanoparticle stability, pharmacokinetics, and targeting (Beach et al., 2024).

### 2.2 Architecture

Architectural diversity underpins the performance of PNs. At the polymer level, linear, branched, star-shaped, and hyperbranched topologies enable precise control of hydrophobic/hydrophilic balance and cargo-loading capacity (Lagarrigue et al., 2023). Amphiphilic block copolymers self-assemble into micelles with hydrophobic cores and hydrophilic shells, well-suited for solubilizing poorly soluble drugs. Polymersomes (vesicle-like structures) mimic liposomes but with greater mechanical stability, allowing encapsulation of both hydrophobic and hydrophilic cargo (Fonseca et al., 2024). Nanogels offer high water content and responsiveness to local stimuli (Blagojevic and Kamaly, 2025), while polyion complexes efficiently condense nucleic acids (Porello et al., 2024). Hybrid architectures combine inorganic cores (gold, silica, iron oxide) with polymer coatings for imaging or theranostic applications (Hosseini et al., 2023). Beyond spherical morphologies, rods, worms, and disks exhibit altered biodistribution and cellular uptake (Lagarrigue et al., 2023). The modularity of polymer chemistry and self-assembly strategies thus enables fine-tuning of nanoparticle architecture to optimize drug loading, release kinetics, and tissue targeting (Beach et al., 2024).

### 2.3 Manufacturing

Multiple fabrication techniques yield PNs with tailored properties. Self-assembly of amphiphilic copolymers produces micelles and vesicles under aqueous conditions (Sarkar and Alexandridis, 2012; Beach et al., 2024). Nanoprecipitation and dialysis methods generate nanoparticles by controlled solvent exchange, enabling reproducibility and scalability (Zielińska et al., 2020). Emulsion-based techniques (single and double emulsion) allow encapsulation of hydrophobic and hydrophilic cargo; emulsion polymerization enables *in situ* particle formation (Zielińska et al., 2020; Beach et al., 2024). Ion gelation/sol–gel methods are widely used for polyion-based nanoparticles, while polymerization-induced self-assembly (PISA) integrates synthesis and self-assembly into a single step (Gu et al., 2023). Spray drying and templated assembly provide solid particles with controlled size and porosity (Hashim and Abdrabou, 2024). Drug incorporation can be passive (during particle formation) or active (post-loading, exploiting pH or ion gradients), the latter improving encapsulation efficiency and release control (Pieper et al., 2019). Various conjugation strategies are employed to graft polymers onto active substances such as drugs, nucleic acids, and proteins, including covalent coupling (e.g., amide, ester, or thiol–maleimide linkages), electrostatic complexation, hydrophobic interactions, and bio-orthogonal click reactions, enabling controlled release, improved stability, and targeted delivery (Moncalvo et al., 2020; Porello et al., 2024). These diverse methods offer flexibility in producing nanoparticles with reproducible physicochemical characteristics, scalable production, and tailored drug release profiles.

### 2.4 Applications

PNs are investigated across a wide therapeutic landscape. In oncology, they enhance tumor targeting via passive accumulation (enhanced permeability and retention, EPR) or active targeting with ligands, improving efficacy while reducing systemic toxicity (Bertrand et al., 2014). In vaccines, polymer nanoparticles enable immune activation through antigen presentation and adjuvant co-delivery (Pippa et al., 2021; Zhang et al., 2023). Antimicrobial therapy benefits from nanoparticles that penetrate biofilms or deliver antibiotics to intracellular pathogens, addressing drug resistance (Naik et al., 2024). For neurological disorders, strategies aim to cross the blood–brain barrier via receptor-mediated transcytosis (Zhang et al., 2021). In cardiovascular and metabolic diseases, polymer carriers provide sustained drug release and reduced dosing frequency (Jha et al., 2024; Gao et al., 2025). Emerging theranostic platforms integrate imaging agents (MRI, PET, phototherapy) with drugs for combined diagnosis and treatment (Hosseini et al., 2023). Despite limited clinical adoption, ongoing trials highlight their promise. The bench-to-bedside development depends on addressing scalability, reproducibility, and regulatory hurdles.

### 2.5 Approved PNs

**FIGURE 1:**
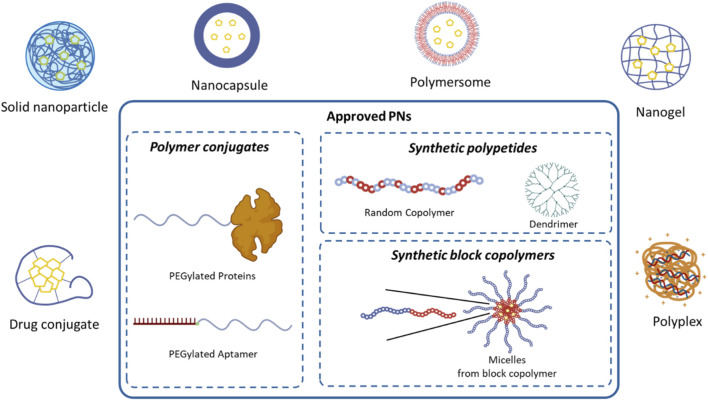
Main classes of PNs under development and a subset of marketed nanomedicines as approved by EMA and/or FDA.

According to the EU-IN Horizon Scanning Report on “Nanotechnology-based medicinal products for human use,” released in 2025 (EMA/HMA, 2025), the PNs marketed as approved by EMA and/or FDA are summarized in Table 1. Among different types of PNs which have been designed and developed for biomedical applications, three main classes of approved PNs can be identified: protein-polymer conjugates (PEGylated therapeutic proteins), drug-loaded PNs made of amphiphilic block copolymers (PEG-polyester block copolymers), and synthetic polypetides (Figure 1).

**TABLE 1:** Overview of authorized PNs as medicinal products by EMA/FDA.

Product name	PN type	Polymer	Active substance	Administration route	Application	Regulator (approval date)	Ref.
Adagen^®^	Protein-polymer conjugate	PEG	bovine adenosine deaminase	Intramuscular injection	Severe combined immunodeficiency disease	FDA (1990)	Moncalvo et al. (2020)
Oncaspar^®^	Protein-polymer conjugate	PEG	L-asparaginase	Intramuscular injection/Intravenous infusion	Acute lymphoblastic leukemia	EMA (2016) FDA (1994)	FDA (2020a), Moncalvo et al. (2020), EMA (2025)
PegIntron^®^	Protein-polymer conjugate	PEG	IFN-α2b	Subcutaneous injection	Hepatitis C	EMA (2000) FDA (2001)	Moncalvo et al. (2020), EMA (2025)
Pegasys^®^	Protein-polymer conjugate	PEG	IFN-α2a	Subcutaneous injection	Hepatitis C	EMA, FDA (2002)	FDA (2020a), Moncalvo et al. (2020), EMA/HMA (2025)
Neulasta^®^	Protein-polymer conjugate	PEG	G-CSF	Subcutaneous injection	Neutropenia during chemotherapy	EMA, FDA (2002)	FDA (2020a), Moncalvo et al. (2020), EMA/HMA (2025)
Somavert^®^	Protein-polymer conjugate	PEG	Engineered hGH	Subcutaneous injection	Acromegaly	EMA (2002) FDA (2003)	FDA (2020a), Moncalvo et al. (2020), EMA/HMA (2025)
Mircera^®^	Protein-polymer conjugate	PEG	Epoetin-β	Subcutaneous/Intravenous injection	Anemia associated with kidney disease	EMA, FDA (2007)	FDA (2020a), Moncalvo et al. (2020), EMA/HMA (2025)
Cimzia^®^	Protein-polymer conjugate	PEG	Fab’ antibody fragment	Subcutaneous injection	Rheumatoid arthritis and Crohn’s disease	EMA (2009) FDA (2008)	FDA (2020a), Moncalvo et al. (2020), EMA/HMA (2025)
Krystexxa^®^	Protein-polymer conjugate	PEG	Uricase	Intravenous infusion	Chronic gout	EMA (Withdrawn) FDA (2010)	Moncalvo et al. (2020), EMA (2025)
Sylatron^®^ (PegIntron)	Protein-polymer conjugate	PEG	INF-α2b	Subcutaneous injection	Melanoma	EMA (Withdrawn) FDA (2001)	FDA (2020a), Moncalvo et al. (2020), EMA (2025)
Lonquex^®^	Protein-polymer conjugate	PEG	G-CSF	Subcutaneous injection	Neutropenia	EMA (2013)	Moncalvo et al. (2020), EMA (2025)
Plegridy^®^	Protein-polymer conjugate	PEG	INF-β1a	Subcutaneous/Intramuscular Injection	Multiple sclerosis	EMA, FDA (2014)	Moncalvo et al. (2020)
Adynovi^®^/Adynovate^®^	Protein-polymer conjugate	PEG	Coagulation factor VIII	Intravenous injection	Hemophilia A	EMA (2018) FDA (2015)	Moncalvo et al. (2020), EMA/HMA (2025)
Refixia^®^/Rebinyn^®^	Protein-polymer conjugate	PEG	Coagulation factor IX	Intravenous injection	Hemophilia B	EMA, FDA (2017)	Moncalvo et al. (2020)
Jivi^®^	Protein-polymer conjugate	PEG	Coagulation factor VIII	Intravenous injection	Hemophilia A	EMA, FDA (2018)	Moncalvo et al. (2020), EMA (2025)
Palynziq^®^	Protein-polymer conjugate	PEG	Phenylalanine ammonia lyase	Subcutaneous injection	Phenylketonuria	EMA (2019) FDA (2018)	FDA (2020a), Moncalvo et al. (2020)
Esperoct^®^	Protein-polymer conjugate	PEG	Coagulation factor VIII	Intravenous injection	Hemophilia A	EMA, FDA (2019)	Moncalvo et al. (2020), EMA (2025)
Macugen^®^	Aptamer-polymer conjugate	PEG	anti-VEGF RNA aptamer	Intravitreal Injection	Choroidal neovascularization	EMA (Withdrawn) FDA (2004)	Halwani (2022)
Copaxone^®^	Synthetic polypetide	Glatiramer acetate	Glatiramer acetate (polypeptide consisting of L-alanine, L- glutamic acid, L-tyrosine, and L-lysine)	Subcutaneous injection	Immunomodulator for multiple sclerosis	FDA (1996) EMA (2001) UK/H/0453	FDA, 1996; EMA/HMA (2025)
VivaGel®	Dendrimer (polypetide)	Poly (L-lysine)-based dendrimer	Astrodimer sodium (Poly (L-lysine)-based dendrimer)	Topical - vaginal	Prevention of recurrent bacterial vaginosis	FDA (2015)	Halwani (2022)
Genexol-PM	Polymer micelles	Mono-methoxy PEG-PDLLA	Paclitaxel	Intravenous injection	Breast cancer	FDA (2007)	Halwani (2022)

**FIGURE 2:**
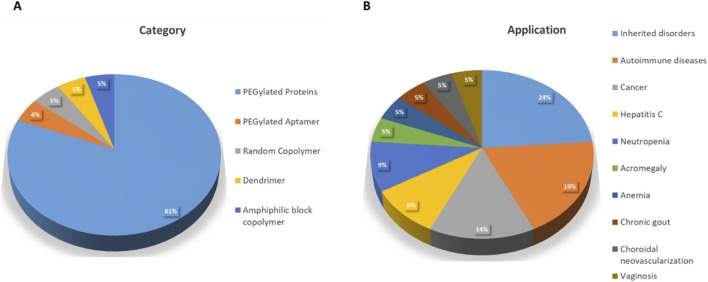
Pie charts representing proportion of different categories of approved PNs **(A)** and their clinical application **(B)**.

From this overview of products, it can be seen that the approved PN are relatively “old” in terms of nanomaterial design and complexity. While in the late 70’–80’s academic and industrial groups explored PEGylation with various proteins showing that attaching PEG chains could increase half-life and reduce immunogenicity (Abuchowski et al., 1977). In the 1990 we see the first FDA-approved PEGylated therapeutic protein (Adagen®, pegademase bovine) (Alconcel et al., 2011). Early preclinical studies in the 1980s–90s showed that amphiphilic block copolymers such as PEG–PLA and PEG–PLGA self-assemble into micelles or nanoparticles, enhancing solubility of hydrophobic drugs and increasing circulation time through PEG hydrophilicity and biodegradability of the polyester block (Kulthe et al., 2012). Genexol-PM®, a PEG-(D,L-lactic acid) micelle formulation of paclitaxel, first approved in South Korea and by FDA in the 2000s, is an example of the earliest polymeric micelle nanomedicine (Kim et al., 2004). A classification of approved PNs and their clinical applications is presented in Figure 2, which highlights the disproportionate dominance of PEGylated proteins among approved PNs. Clinically, most products target oncology or chronic diseases, underscoring how regulatory familiarity and established safety profiles strongly influence which PN platforms achieve approval. These trends also correlate with their clinical indications and routes of administration. PEG–protein conjugates, used mainly in oncology, hematological disorders, and chronic inflammatory diseases, are almost exclusively delivered via parenteral administration, which bypasses major biological barriers and ensure predictable systemic exposure. Overall, the distribution of PN types, administration routes, and therapeutic fields underscores how biological barriers and immune responses shape which PNs successfully reach the clinic.

### 2.6 Current limitations

Despite the vast chemical and structural possibilities offered by PNs, the list of regulatory approvals remains strikingly short, dominated by PEGylated proteins, a few synthetic polypeptides, and relatively simple PEG–polyester block copolymers. This contrast between laboratory research and clinical reality underscores several bottlenecks, which fall into three main categories: scientific gaps, manufacturing and characterization challenges, and regulatory consequences, as detailed below.

#### 2.6.1 Scientific gaps

Regulators have consistently favored polymers with established safety profiles, such as PEG and biocompatible polyesters, because their physicochemical properties, biodistribution, and toxicology are well documented. More sophisticated constructs such as hyperbranched scaffolds, stimuli-responsive systems, or polymer hybrids, raise too many unanswered questions about stability, immunogenicity, and long-term effects (Eltaib, 2025). Immunological barriers add to the challenge: PEG, once the gold standard, now suffers from anti-PEG antibodies and accelerated clearance, while many cationic polymers remain clinically untenable due to toxicity (Moncalvo et al., 2020). Perhaps the most sobering bottleneck lies in biology itself. Promising animal results are rarely replicated in humans, where tumors are less leaky, physiological barriers more stringent, and patient populations highly heterogeneous. The much-celebrated EPR effect, for example, has proven inconsistent and often negligible in clinical settings (Nakamura et al., 2016).

Active targeting also faces major hurdles: protein corona masking, heterogeneous receptor expression, poor tissue penetration, endosomal trapping, and competition with endogenous ligands (Rampado et al., 2020). These factors mean ligand-based strategies rarely yield substantial improvements over passive delivery, explaining why clinical gains have been modest despite strong preclinical promise.

#### 2.6.2 Manufacturing and characterization challenges

At the same time, industrial implementation remains a weak point: elegant laboratory protocols such as controlled/living polymerizations, nanoprecipitation or dialysis rarely scale reliably, and their sensitivity to subtle parameters often leads to poor batch-to-batch reproducibility. These limitations are further amplified under Good Manufacturing Practice (GMP) constrains, where process robustness, sterility, and consistent product quality are mandatory but difficult to maintain for complex nanosystems. Even minor variations in particle size or drug loading can jeopardize approval (Souto et al., 2020).

A lack of standardized, validated methods to reliably measure nanoscale properties, such as surface characteristics, protein corona formation, stability in biological media, and drug loading/release, combined with the difficulty of linking these measurements to *in vivo* behavior, further complicates physicochemical characterization and comparability assessments. Finally, regulators demand pharmacokinetic predictability, yet sophisticated responsive systems, designed to release drugs under pH, enzyme, or redox triggers, can behave unpredictably across patients.

#### 2.6.3 Regulatory consequences

The cumulative effect is a regulatory and industrial environment that rewards simplicity and penalizes innovation. Progress in the field requires directly addressing these systemic hurdles: harmonized standards for nanoparticle characterization, scalable and reproducible manufacturing methods, and rigorous human data to validate translational relevance. Without such advances, most of the PN design will remain an academic exercise with limited clinical impact.

Furthermore, the absence of nanomedicine-specific regulations across agencies means that PNs are assessed under conventional frameworks, resulting in case-by-case evaluations that contribute to fragmented expectations, inconsistent data requirements, and uncertainty in approval timelines.

Collectively, the limitations described above illustrate why traditional regulatory models are often insufficient for PNs. In comparison to conventional pharmaceutical products, most PNs are complex in nature, where more than one component can affect the pharmacological behavior of the active ingredient (Halwani, 2022). Due to this complexity and unique category of therapeutic agents, the regulation of this type of nanomedicines may face several obstacles, and in general, there is a lack of standards in the evaluation processes (Desai, 2012; Mühlebach, 2018).

## 3 Regulatory framework

Currently, new medicines based on nanotechnology are evaluated by the US Food and Drug Administration (FDA), the European Medicines Agency (EMA), and other agencies including Health Canada (HC, Canada), Pharmaceuticals and Medical Devices Agency (PMDA, Japan) under the Japanese Ministry of Health, Labour and Welfare (MHLW), and National Medical Products Administration (NMPA, China) (Halwani, 2022; Clogston et al., 2024), Medicines and Healthcare products Regulatory Agency (MHRA, United Kingdom) (MHRA, 2025). These regulatory authorities generally use a case-by-case approach under the traditional framework of benefit/risk analysis (Desai, 2012; Soares et al., 2018; Halwani, 2022). Nanotechnology-based health products are regulated under existing frameworks for medicinal products or medical devices, depending on their primary mode of action. However, due to their unique nanoscale properties, regulatory authorities require additional characterization, as outlined in recent guidance documents (Halamoda-Kenzaoui et al., 2021), as summarized in Table 2.

**TABLE 2:** Regulatory EMA/FDA documents specifically addressing nanotechnology-based health products, which include PNs.

Category	Examples	Documents
Products of nanotechnologies	Health products (as well as electronics, cosmetics, textiles).	• SCENIHR. Risk assessment of products of nanotechnologies. (SCENIHR, 2009) • FDA. Considering Whether an FDA-Regulated Product Involves the Application of Nanotechnology. (FDA, 2014a)
Nanotechnology-based medicinal products	Copaxone (Glatinamer acetate) VivaGel (PLL-based dendrimer)	• EMA. Nanotechnology-based medicinal products for Human Use (EMA/HMA, 2025) • EMA. Reflection paper on nanotechnology-based medicinal products for Human Use. (EMEA/CHMP, 2006) • FDA. Guidance for Industry: Drug Products, including Biological Products, that Contain Nanomaterials. (FDA, 2022)
Block copolymer micelles	Genexol-PM (PEG-PDLLA)	• Joint MHLW/EMA reflection paper on the development of block copolymer micelle medicinal products. (EMA, 2013a)
Coated nanomedicines	PEGylated liposomes, nanomedicine products with targeting ligands	• EMA. Reflection paper on surface coatings: general issues for consideration regarding parenteral administration of coated nanomedicine products. London. (EMA, 2013b) • EMA. Reflection paper on the data requirements for intravenous liposomal products developed with reference to an innovator liposomal product (EMA, 2013c) • FDA. Liposome Drug Products Chemistry, Manufacturing, and Controls; Human Pharmacokinetics and Bioavailability; and Labeling Documentation Guidance for Industry. (FDA, 2018c)
Protein-polymer conjugates	PEGylated proteins	• EMA. Guidance on the Description of the Composition of Pegylated (Conjugated) Proteins in the SPC (CPMP/BWP/3068/03) (EMA, 2003) • EMA. Concept paper on potency declaration/labelling for biological medicinal products which contain modified proteins as active substance. (EMA, 2011a) • EMA. CHMP Safety Working Party’s Response to the PDCO Regarding the Use of PEGylated Drug Products in the Paediatric Population. (EMA/CHMP/SWP/647258/2012) (EMA, 2012) • FDA. Guidance for Industry: Immunogenicity Assessment for Therapeutic Protein Products. (FDA, 2014b) • FDA. Guidance for Industry: Assay Development and Validation for Immunogenicity Testing of Therapeutic Protein Products. (FDA, 2016)

The EMA regulates nanomedicines under the same frameworks as conventional medicinal products, including biologicals, small molecules, and advanced therapy medicinal products (ATMPs), without a dedicated regulatory pathway. They are evaluated under Directive 2001/83/EC on Medicinal Products for Human Use (EC, 2001), which governs the EU marketing authorization process and is supported by additional Directives, Commission regulations, and various legal reference documents. Evaluation covers preclinical studies, GMP compliance, quality documentation, and clinical performance to ensure safety, efficacy, and consistency. While no specific EU framework exists for nanomedicines, the EMA has issued reflection papers offering preliminary guidance on their development and evaluation (EMA, 2008b; EMA, 2011b; EMA, 2013a; EMA, 2013b; EMA, 2016), offering recommendations for preparing marketing authorization applications (Soares et al., 2018). Although these documents are not legally binding, they indicate EMA expectations and provide a basis for dialogue between developers and regulators. Key documents include:

- Reflection Paper on Block Copolymer Micelle Products (2013): Relevant for amphiphilic polymeric nanocarriers, this guidance discusses the need for rigorous analysis of micelle formation, stability, and potential release of unbound polymeric species (EMA, 2013a).
- Reflection Paper on Surface Coatings of Nanomedicines (2021): Recognizing that surface modifications (e.g., PEGylation, ligand conjugation) dramatically influence pharmacokinetics, immunogenicity, and biodistribution, this paper outlines how such attributes must be characterized and justified throughout development (EMA, 2013b).

Other reflection papers on iron colloids (EMA, 2015; EMA, 2011b), liposomes and other lipid-based nanocarriers (EMA, 2013c), while not specific to PNs, highlight critical aspects of physicochemical characterization, stability, and biological fate applicable across nanomedicine classes.

The Nanotechnology-based medicinal products for human use EU-IN Horizon Scanning Report (EMA/HMA, 2025), reviews nanotechnology-based medicinal products, highlighting trends, applications in drug delivery, and potential in cancer and infectious disease treatment. It stresses the need for strong regulatory frameworks and early identification of innovations to guide EMA in ensuring product quality, safety, efficacy, and future readiness.

In terms of definitions and classifications, the EU first established a definition for nanomaterials in 2011, identifying them as materials with dimensions between 1 and 100 nm (EC, 2011). This definition was revised in 2022 to encompass more complex nanoscale systems (EC, 2022). For polymeric nanocarriers typically ranging from 20 to 200 nm, this classification entails additional regulatory requirements, including advanced physicochemical characterization and comprehensive toxicological assessments (ECHA, 2022; JRC/EU, 2022; Bleeker et al., 2023).

The EMA assesses PNs as either biological or nonbiological medicinal products, with classification determined by the active substance and primary mechanism of action (Hussaarts et al., 2017). Both categories require evaluation beyond plasma pharmacokinetics, including stepwise comparison with a reference product to establish bioequivalence, safety, quality, and therapeutic efficacy (Astier et al., 2017; Soares et al., 2018). Regulatory approaches for nonbiological complex drugs (NBCDs) often draw from biological frameworks, as both involve incomplete structural characterization and manufacturing-dependent *in vivo* activity, thereby necessitating ongoing comparability assessments (Schellekens et al., 2014; Soares et al., 2018).

The FDA regulates pharmaceutical products under two main laws: the Federal Food, Drug, and Cosmetic (FDA, 2018a) and the Public Health Service Act, which covers biologically derived therapies (Mühlebach, 2018; Halwani, 2022). These laws classify products by primary mode of action: chemical (drug), mechanical (device), or biological (Mühlebach, 2018).

Nanomedicines are treated as complex products and not categorically deemed safe or harmful; each is evaluated individually. Since December 2016, FDA procedures for classifying and assessing combination products (those combining drugs, devices, and/or biologics) have included nanomedicines (Mühlebach, 2018). Examples include FDA-approved nanoformulations of paclitaxel and doxorubicin for cancer treatment.

Concerns remain over nanoparticle toxicity, ability to cross the blood–brain barrier, and long-term effects (Bawa and Johnson, 2007; Resnik and Tinkle, 2007; Mühlebach, 2018). In the last years, the FDA has issued specific guidance documents on nanotechnology and nanomedicines, urging manufacturers to consult the agency before marketing (FDA, 2014a; FDA, 2018a; FDA, 2018d; FDA, 2018c; FDA, 2020c).

In 2017, the FDA released draft guidance on requirements for drug products, including biologics, containing nanomaterials (FDA, 2017). The final version of this guidance was issued in April 2022 (FDA, 2022).

Regarding PNs, guidance documents and reflection papers have been provided by EMA, FDA and other international regulatory authorities, specifically EMA/MHLW for block-copolymer-micelle medicinal products (EMA, 2013a), and MHLW for nucleic acid (siRNA)-loaded nanotechnology-based drug products (MHLW, 2016).

For medical devices containing nanomaterials, guidance has been issued by the Scientific Committee on Emerging and Newly Identified Health Risks (SCENIHR) (EC, 2015) and the International Organization for Standardization (ISO) (ISO, 2012).

More references are reported in Table 2. These documents outline specific nanotechnology-related properties that may influence product quality, safety, and efficacy.

In summary, despite differences in terminology and procedural steps, regulatory authorities share a common principle whereby nanomedicines, including PNs, are evaluated under the same legal frameworks that govern conventional pharmaceuticals. Neither EMA nor FDA agency applies nanomedicine-specific approval pathways; instead, nanoscale considerations are integrated into existing drug and biologic evaluations. EMA Reflection papers (EMEA/CHMP, 2006; EMA, 2013a; EMA, 2013b; EMA, 2013c; EMA/HMA, 2025) and the FDA Nanomaterials Guidance (FDA, 2022) consistently note that current tools are adaptations of traditional pharmaceutical assessment approaches. This results in heterogeneous, product-specific data requests and regulatory uncertainty, especially for emerging polymeric architectures whose physicochemical characterization, surface interactions, and biological fate are more complex than conventional dosage forms. Developers therefore face unpredictability in meeting expectations for comparability, stability, and clinical performance.

The FDA Nanotechnology Task Force, created to assess emerging scientific and regulatory challenges, reported in the Nanotechnology Task Force Report (FDA, 2007) that limited understanding of nanomaterial behavior, inadequate testing methods, and unclear regulatory pathways required stronger scientific capacity and clearer guidance to ensure product safety. More recently, the report “The Nanotechnology-Over a Decade of Progress and Innovation” (FDA, 2020b) details FDA’s progress, including expanded research infrastructure, improved staff training, strengthened collaborations, and development of standards, thereby highlighting the benefits of a science-based, product-focused regulatory approach supporting innovation while maintaining safety oversight.

## 4 Regulatory information required

Regulatory agencies demand rigorous, multi-method validation of nanoscale properties, tied directly to manufacturing control and therapeutic function. Collectively, the guidance documents emphasize characterization needs that extend beyond conventional requirements, including detailed physicochemical and biological assessments. Key considerations include stability, sterility, pharmacokinetic and pharmacodynamic behavior, and bioburden. While not stand-alone requirements, these parameters complement existing regulatory guidelines, addressing the unique challenges posed by nanotechnology-based products, particularly in the context of drug delivery systems (JRC/EC, 2019; Halamoda-Kenzaoui et al., 2021; Floyd et al., 2025). The regulatory information required to nanotechnology-based health products, specifically PNs, is summarized in Table 3.

**TABLE 3:** Regulatory information required for polymer nanoparticles as nanotechnology-based health products (JRC/EC, 2019; Halamoda-Kenzaoui et al., 2021; Floyd et al., 2025), corresponding CQAs and recommended techniques for characterization.

Category	Information required	Corresponding CQAs	Recommended analytical techniques
Physicochemical	Chemical composition and structure	Polymer identity, monomer ratio, molecular weight (Mn, Mw), dispersity (Đ)	NMR, FTIR, SEC, MS
	Functional structural attributes	Polymer architecture (linear, branched, crosslinked), functional groups, copolymer composition	NMR, FTIR, SEC, HPLC, SAXS, TEM
	Impurities	Residual monomers, catalysts, solvents, unreacted reagents, degradation products	HPLC, GC-MS, LC-MS, ICP-MS
	Particle size distribution, shape, morphology	Particle size (mean, PDI), morphology, aspect ratio	DLS, NTA, (cryo)TEM, SEM, AFM
	Surface properties	Zeta potential, surface chemistry, surface ligand density, PEG coverage, hydrophobicity index	ELS, XPS, ATR-FTIR, contact angle, colorimetric assays
	Internal structure	Porosity, internal architecture, encapsulation domain	BET, cryo-TEM, SAXS/SANS, AFM
	Particle concentration	Particle number per mL, mass per volume	DLS/NTA, UV–Vis, gravimetric
	Degradation pathway, kinetics, products	Polymer degradation rate, mechanism, degradation product identity	HPLC/LC-MS, SEC, DSC/TGA, FTIR, hydrolysis studies
	Stability (physical and chemical under relevant conditions)	Stability in storage and physiological media (size, charge, drug retention)	DLS/time-course, ELS, HPLC, (cryo)TEM, DSC
Drug delivery–specific	Drug loading efficiency	Encapsulation efficiency (EE%), drug loading (DL%)	HPLC/UPLC, LC-MS, UV–Vis, fluorescence spectroscopy, AF4
	Distribution of active ingredient (bound vs. free)	Free vs. encapsulated drug fraction, payload homogeneity	Ultrafiltration, centrifugation, HPLC, SEC, AF4, LC-MS
	Physical state of active substance	Crystalline vs. amorphous, molecular dispersion	XRD, DSC, (solid-state) NMR, FTIR
	*In vitro* release rate (drug/nucleic acid in relevant media)	Release kinetics, mechanism of release	Dialysis, sample-and-separate, HPLC/LC-MS, UV–Vis
Biological and pharmacokinetics	Bioburden: sterility and endotoxins	Sterility, endotoxin level, *mycoplasma* contamination	Sterility tests (Ph. Eur./USP), LAL, qPCR for *mycoplasma*
	Stability in blood/Serum	Colloidal stability, drug retention, aggregation in serum	DLS, SEC-HPLC, protein binding assay, TEM
	Biological fate and accumulation	Biodistribution, tissue accumulation, clearance pathway	Radiolabeling, fluorescence/MRI tracking, LC-MS, qPCR
	ADME profile	Absorption, distribution, metabolism, excretion	HPLC-MS/MS, LC–HRMS, qPCR
	Protein binding/Corona formation	Protein corona identity and stability	SDS-PAGE, LC-MS/MS, DLS
	*In vivo* degradation/Solubilisation rate and site	Degradation kinetics, biodegradation products, clearance organs	LC-MS, histology, PET/MRI tracking
Pharmacodynamics and safety	Biocompatibility with blood/Serum	Hemolysis potential, complement activation, cytokine response	Hemolysis assay, (CH50, SC5b-9) ELISA, DLS/zeta-potential, cytokine panel
	Risks by administration route	Local toxicity, irritation, injection site reaction	*In vivo* irritation tests (OECD, ISO), histopathology, local tolerance studies
	Uptake and cytotoxicity in phagocytes	Cellular uptake efficiency, lysosomal accumulation, cytotoxicity	Flow cytometry, confocal microscopy, MTT/XTT, LDH
	Interaction with enzymes	Enzymatic degradation, inhibition of metabolic enzymes	HPLC, enzyme inhibition kinetics, spectrophotometric assays, LC-MS for metabolites
	Immunogenicity	Cytokine induction, antibody formation, immune cell activation	PBMC cytokine release, ELISA (ADA), TLR activation assay, complement activation

### 4.1 Analytical techniques for physicochemical characterization

Core chemical and structural analyses rely on nuclear magnetic resonance (NMR), infrared spectroscopy (ATR-FTIR), size exclusion chromatography (SEC), high-performance liquid chromatography (HPLC), and mass spectrometry (MS) to determine molecular weight, composition, functionalization, and drug release kinetics. Microscopy technique, particularly transmission electron microscopy (TEM and cryo-TEM), scanning electron microscopy (SEM), and atomic force microscopy (AFM), enable nanoscale imaging of morphology, internal structure and porosity, and dynamic transformations. Light scattering (DLS, NTA) and small-angle scattering (SAXS, SANS) provide information on particle size distribution, aggregation number, and assembly mechanisms in solution, often with real-time monitoring capabilities (Floyd et al., 2025).

Zeta potential indicates surface charge and colloidal stability, directly influencing circulation, aggregation, and biodistribution, and it is typically measured by Electrophoretic Light Scattering (ELS). For porous nanomaterials, porosity governs drug loading, encapsulation efficiency, and release control. Both require standardized, reproducible measurement methods for regulatory compliance.

### 4.2 Drug loading and release studies

Drug loading and release profiles are central to therapeutic efficacy. HPLC (Tsarenko et al., 2023), dialysis (Mast et al., 2021), and asymmetrical flow field-flow fractionation (AF4) (Shakiba et al., 2021) are widely used to quantify encapsulated versus released drug, with AF4 offering improved resolution of size-dependent release behavior. These methods must be validated for reliability, as premature release or incomplete cargo delivery undermines safety and efficacy.

### 4.3 Biological and pharmacokinetics

Detailed biological and pharmacokinetic characterization is required to ensure safety and efficacy. Sterility and endotoxin levels are assessed through bioburden and Limulus Amebocyte Lysate (LAL) assays (Hannon and Prina-Mello, 2021), while stability in blood or serum is tested using DLS and zeta potential analysis to monitor aggregation and degradation (Lazzari et al., 2012). Biodistribution and accumulation are evaluated with fluorescence or radiolabel imaging, to quantify organ uptake (Veronesi et al., 2017). The ADME profile of both carrier and drug is determined using HPLC-MS/MS and *in vivo* pharmacokinetic studies (Šimek et al., 2019), complemented by tissue histopathology. Protein corona formation is analyzed by SDS-PAGE, LC-MS proteomics (Partikel et al., 2019), while polymer degradation and clearance are characterized through SEC, NMR, and MS of biological samples (Floyd et al., 2025). Together, these techniques provide a comprehensive understanding of PN behavior *in vivo*, guiding regulatory compliance and formulation optimization.

### 4.4 Pharmacodynamics and safety

Pharmacodynamic and safety testing of PNs assesses their biological compatibility and potential immunotoxicity. Hemocompatibility and serum stability are evaluated using hemolysis assays, complement activation (CH50, SC5b-9 ELISA), and DLS or zeta potential measurements to monitor aggregation in plasma (Dobrovolskaia and McNeil, 2007). Cellular uptake and cytotoxicity in macrophages or other phagocytes are quantified by flow cytometry, confocal microscopy, and MTT/XTT or LDH assays (Toscano and Torres-Arias, 2023). Enzyme interaction studies, performed via HPLC, enzyme inhibition kinetics, or spectrophotometric assays, reveal potential interference with metabolic or lysosomal enzymes (Nel et al., 2009). Immunogenicity, following ICH S8 guidance, is assessed using cytokine panels (ELISA, multiplex bead assays) and anti-polymer antibody ELISAs, complemented by *in vivo* cytokine profiling (Dobrovolskaia, 2022). Together, these techniques ensure PNs meet regulatory expectations for systemic safety and immune compatibility.

### 4.5 Critical quality attributes

Regulatory bodies such as EMA and FDA require systematic evaluation of critical quality attributes (CQAs), as part of pharmaceutical quality assessment under the International Conference on Harmonisation of Technical Requirements for Registration of Pharmaceuticals for Human Use considerations (ICH), guideline Q8 (R2) on pharmaceutical development (EMA, 2006), Q9 on quality risk management (EMA, 2023), and Q10 on pharmaceutical quality system (EMA, 2008a).

CQAs for PNs include particle size, morphology, surface charge, porosity, stability, drug loading, and release kinetics (Table 3). For nanomedicines, EMA’s reflection papers and FDA guidances explicitly mention particle size, morphology, surface charge, and stability as key CQAs.

Quality by Design (QbD), as outlined in ICH Q8 (R2), is a systematic, science- and risk-based approach to pharmaceutical development that emphasizes building quality into the product from the outset rather than relying on end-point testing. Within the QbD framework, CQAs are systematically linked to critical process parameters (CPPs) and material attributes (CMAs) to define a design space that ensures consistent product quality. This approach is explicitly endorsed by both the FDA and EMA for nanotechnology-based formulations.

### 4.6 Manufacturing and regulatory controls

The manufacturing and regulatory control of PNs focuses on ensuring consistent, reproducible product quality, safety, and efficacy during large-scale production. Both EMA and FDA apply a risk-based QbD approach, emphasizing the control of CPPs that directly influence CQAs. Manufacturing must be performed under GMP with validated unit operations, in-process controls, and monitoring to maintain batch-to-batch consistency and product stability. Regulators require comprehensive documentation of the manufacturing process in the Common Technical Document (CTD, Module 3) (FDA, 2001; EMA, 2021), including process validation, scale-up rationale, control strategies, and evidence of reproducibility. The EMA references its reflection papers and guidance on nanotechnology-based products (EMEA/CHMP, 2006; EMA/HMA, 2025), while the FDA provides guidance on *Drug Products, Including Biological Products, that Contain Nanomaterials* (FDA, 2017). Both agencies mandate validated analytical methods for in-process and release testing, stability monitoring, and control of impurities or degradation products. The overarching goal is to ensure that the manufactured nanotherapeutic consistently meets defined quality specifications, independent of the initial polymer design.

### 4.7 Clinical trials

Clinical trials for new medicines are conducted in three phases, to evaluate safety, efficacy, pharmacokinetics, and side effects in progressively larger human populations, continuing into a fourth phase of post-marketing surveillance to monitor long-term safety. Clinical trial phases for PNs follow the same structural framework as conventional pharmaceuticals, although their development requires more extensive preclinical and clinical evaluation (Souto et al., 2020). Before human testing, both the EMA and FDA require pivotal nonclinical safety and toxicology studies to comply with Good Laboratory Practice (GLP) standards (EMA, 2004; CFR, 2025). Both regulators emphasize that nanomaterial-containing products must meet the same quality and safety standards as conventional drugs, but with additional attention to nanomaterial-specific risks such as biodistribution, persistence, and immune activation (Dobrovolskaia et al., 2008; Dobrovolskaia, 2015).

Over the last decade, European legislation, notably Directive 2001/20/EC, aligned clinical trial requirements across Member States, enforcing Good Clinical Practices (GCP) through ICH guidelines. The EMA’s EudraLex codifies regulations for medicinal product development, while the EudraCT database standardizes trial reporting, streamlining R&D and enhancing transparency and competitiveness in the pharmaceutical industry (Souto et al., 2020). Clinical trials in the U.S. are regulated under the Title 21 of the Code of Federal Regulations (CFR), ensuring ethical conduct (CFR, 2025). The FDA also adopts ICH E6 (R2) GCP guidelines, unified internationally, as the standard for clinical trials (FDA, 2018b).

During clinical trials, PNs undergo enhanced safety monitoring due to the potential immunological and pharmacokinetic complexities of their carriers. Unlike small molecules, nanoparticle formulations can provoke immune responses, including complement activation–related pseudoallergy (CARPA) (Szebeni, 2014; La-Beck et al., 2021), cytokine release syndrome, and anti-polymer antibody formation (Mohamed et al., 2019). Consequently, regulators often require sentinel dosing, controlled infusion rates, and real-time monitoring of cytokine panels and complement split products. Nanoparticles also exhibit distinct biodistribution patterns, commonly accumulating in the liver, spleen, and kidneys (Åslund et al., 2022); this necessitates extended pharmacokinetic studies and frequent assessment of hepatic and renal function. Both the carrier and the released drug must be tracked to verify appropriate clearance and therapeutic release. Developers must track both the nanoparticle carrier and the released drug to ensure proper clearance and therapeutic release. Examples include side effects in clinical trials of other nanomedicines, such as infusion reactions with liposomal doxorubicin (Doxil) (Gabizon et al., 2025), anti-PEG antibodies in patisiran (Onpattro) trials (EMA, 2018), and high hepatic uptake in lipid nanoparticle siRNA therapies (Hosseini-Kharat et al., 2025). These risks necessitate additional imaging, biomarker analyses, and long-term safety follow-up, distinguishing nanomedicine trials from standard drug evaluations (Sudheesh et al., 2022). Such findings have led to stricter post-marketing surveillance and long-term follow-up requirements compared with conventional drugs.

## 5 Methodological gaps

The development and regulatory acceptance of PNs require validated analytical methods that capture their distinct physicochemical and biological characteristics. Despite progress in standardisation, major methodological gaps persist in areas such as polymer characterization, physicochemical properties of the nanocarrier, drug loading and release, behavior in biological media, pharmacokinetics, and immune interactions. Current ISO and ASTM standards remain biased toward classical physicochemical metrics, while neglecting nanospecific attributes such as stability in complex fluids and immune compatibility. Addressing these gaps is essential for regulatory confidence and scientific reproducibility.

Surface properties largely determine nanoparticle behavior in biological environments, influencing protein binding, membrane interaction, and immune recognition (Díez-Pascual, 2022; Ly et al., 2024; Zheng et al., 2025). While standards exist for charge measurement, there are no validated methods for assessing hydrophobicity, ligand density, or coating uniformity. Many nanocarriers rely on polymer coatings such as PEG for stability or targeting, yet current bulk analyses fail to capture particle heterogeneity. Advanced techniques such as atomic force microscopy (AFM), hydrophobic interaction chromatography, and affinity-based binding assays, show research potential but lack regulatory maturity. Even surface area measurement remains problematic; traditional BET gas adsorption applies only to dry powders, not to polymeric nanoparticles in aqueous suspension (Joudeh and Linke, 2022). Thus, reliable characterization of surface attributes remains one of the greatest unmet needs.

Drug loading and release represent the core functional aspects of nanomedicines. Although total drug content can often be quantified by dissolving the carrier followed by chromatographic analysis, determining the fraction of encapsulated versus free drug is technically challenging. Separation techniques such as ultrafiltration risk disrupting the drug–carrier equilibrium, and validated methods are limited to specific formulations. For complex APIs such as mRNA or oligonucleotides, quantification and impurity detection remain under development (Camperi et al., 2024). Standardized assays capable of evaluating release kinetics and carrier integrity across nanocarrier classes are urgently needed to enable meaningful comparisons of therapeutic performance.

Kinetic behavior in biological media presents further complexity. Nanocarriers must be evaluated for both chemical and physical stability, as well as for protein interactions leading to “corona” formation. Measuring release and aggregation in plasma is difficult due to interference from abundant proteins. Methods such as AF4 improve resolution but cannot reliably analyze small organic nanoparticles under 30 nm (Babick et al., 2016). Protein corona analysis, typically using gel electrophoresis and mass spectrometry, applies only to separable particles and lacks consensus on plasma composition, limiting reproducibility and regulatory applicability (Hajipour et al., 2023).

Absorption, distribution, metabolism, and excretion (ADME) studies determine nanoparticle fate *in vivo* (Mitchell et al., 2021). Detecting intact nanocarriers remains challenging, as conventional bioanalytical methods generally measure only released APIs. Metal-based or fluorescently labeled particles allow limited tracking, while most organic systems lack suitable markers. *In vitro* absorption models like Caco-2 cells poorly represent non-oral nanocarriers, and intracellular trafficking assays remain underused (Donahue et al., 2019). Animal models provide essential biodistribution data but often fail to predict human outcomes. Physiologically based pharmacokinetic (PBPK) models could integrate *in vitro* and *in vivo* results to reduce animal testing, yet current models require further validation and standardisation before regulatory implementation (Loisios-Konstantinidis and Dressman, 2021).

Interactions with the immune and circulatory systems represent perhaps the most complex domain. Endotoxin contamination can distort toxicity results, yet standard LAL assays are prone to nanoparticle interference and cannot detect encapsulated endotoxins. Alternative monocyte activation tests provide partial solutions but lack specificity. Hemocompatibility assays, including hemolysis and coagulation studies, are used to predict thrombogenic potential, though none are universally validated (Loisios-Konstantinidis and Dressman, 2021). Immune hypersensitivity reactions such as CARPA are well documented but remain difficult to model accurately *in vitro* (La-Beck et al., 2021; Saxena et al., 2025). Broader innate immune effects (macrophage activation, inflammasome signaling, oxidative stress) are measurable only with assays still at the research stage (Dobrovolskaia, 2022). Adaptive immune assessments, including anti-polymer antibody formation, remain reliant on *in vivo* testing, as no *in vitro* model can yet reproduce human immune complexity.

In summary, analytical methods for PNs evaluation remain fragmented, often adapted from conventional pharmaceutical testing rather than designed for nanoscale systems. Surface characterization, drug release kinetics, in-plasma stability, biodistribution, and immunological assessments all lack standardized, validated procedures. Progress will depend on coordinated collaboration among researchers, regulators, and standardisation bodies to unified protocols, validate emerging analytical techniques, and bridge the gap between experimental capability and regulatory need.

International harmonization efforts, notably the OECD Working Party on Manufactured Nanomaterials and its associated guidance documents, have advanced standardized approaches for nanoscale characterization and safety testing, providing an important foundation for regulatory convergence relevant to nanomedicine (OECD, 2025).

## 6 Future perspective

The recent success COVID-19 nanovaccines is expected to catalyze rapid growth in nanomedicine across oncology, infectious diseases, rare disorders, and beyond (Friedrichs and Bowman, 2021). This progress will bring more frequent and complex regulatory submissions, requiring closer collaboration between industry and regulators to establish best practices for development, presentation, and characterization (Clogston et al., 2024). Ultimately, the future of nanomedicine rests on sustained innovation, cohesive regulatory frameworks, and a shared commitment to delivering safe and transformative therapies to patients worldwide (Halamoda-Kenzaoui et al., 2022).

Advancing analytical methods for precise characterization of PNs is essential to address increasing product complexity, alongside transparent and collaborative engagement with regulatory authorities to ensure successful submissions (Clogston et al., 2024; Floyd et al., 2025). PNs are complex, multicomponent systems in which small variations in synthesis parameters, such as polymer concentration, solvent ratio, mixing speed, or temperature, can markedly influence CQAs such as particle size, surface charge, and drug loading or release profiles.

Since these properties dictate efficacy, safety, and biodistribution, regulatory authorities (FDA, EMA, ICH) increasingly emphasize the use of Process Analytical Technologies (PATs) for real-time process monitoring and control (Gerzon et al., 2022; Floyd et al., 2025). PAT tools such as online light scattering and spectroscopic methods, enable detection of changes in particle formation, solvent removal, and drug encapsulation kinetics. This can facilitate immediate process adjustments, ensuring reproducibility, batch consistency, and compliance with GMP standards, while supporting regulatory expectations for robust QbD manufacturing.

A key future challenge in regulating PNs is the systematic definition of nanomedicine-specific quality attributes (nano-CQAs) that capture nanoscale behavior. Unlike conventional drugs, nano-CQAs such as size-dependent biodistribution, dynamic surface transformations, protein corona formation, and sensitivity to manufacturing variability, are not fully addressed by traditional CQAs (Taha et al., 2020; Dri et al., 2023). Future regulatory approaches should therefore integrate attributes linking nanoscale structure, biological interactions, and *in vivo* performance. Standardized and context-relevant testing methods are essential to ensure reproducible and clinically meaningful characterization of key nanoscale properties. Measurements should combine complementary techniques and be performed under physiologically relevant conditions, enabling comparability across laboratories and development stages.

In nanomedicine design and development, a Safe-by-Design (SbD) approach should be tailored for PNs (Schmutz et al., 2020). Building on general SbD principles from EU initiatives such as NANoREG, NanoReg2, and ProSafe, this framework adapts them to the specific requirements of nanomedicine (Soeteman-Hernandez et al., 2019). SbD aims to identify and mitigate risks early in product development, contrasting with conventional risk assessment applied post-design. While widely used in engineering and conceptually linked to QbD in pharmaceuticals, SbD remains absent from formal regulatory guidelines (ICH, EMA, FDA) (Schmutz et al., 2020).

**FIGURE 3:**
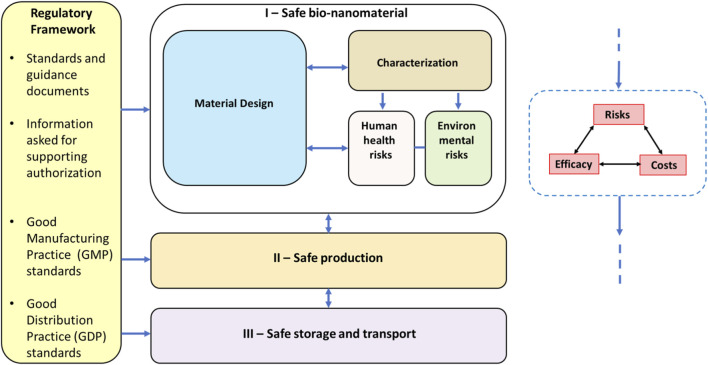
Flow of PNs development process from design to storage and distribution, according to the SbD principles. Any SbD actions taken during the flow are planned to minimize risks and costs, while maintain efficacy (right box).

Currently, PNs lack a systematic approach to incorporate safety throughout the innovation pipeline. The GoNanoBioMat framework (EMPA, 2020) positions SbD as an iterative, feedback-driven process that replaces the linear stage-gate model, integrating safety from concept to commercialization. Built on the three pillars Safe Nanobiomaterials, Safe Production, and Safe Storage and Transport (Figure 3), it promotes innovation efficiency and alignment among industry, regulators, and academia. The process spans Material Design, Characterization, Human and Environmental Risk Assessment, Manufacturing and Control, and Storage and Transport, ensuring regulatory alignment at each stage. Material Design defines intended use and predicts toxicity using literature data and modeling tools (e.g., QSAR, OECD, 2007), though experimental validation remains necessary due to assay limitations. Characterization links polymer and nanoscale traits to biological outcomes, while Risk Assessment evaluates human and environmental hazards. Manufacturing ensures GMP compliance and control of critical attributes. Iterative decision points balance safety, efficacy, and cost, optimizing regulatory-ready PNs. Since absolute safety is unattainable, SbD should be viewed as a strategy to minimize risks while maintaining efficacy. Although not legally binding, it provides guidance for coordinating interdisciplinary collaboration across PN stakeholders. Future expansions should integrate preclinical and clinical testing according to GLP and GCP standards (Schmutz et al., 2020).

Emerging technologies such as artificial intelligence (AI), machine learning (ML), and advanced computational modeling are transforming the development of PNs (Chou et al., 2025). These tools enable the integration and interpretation of complex, multidimensional datasets generated during formulation, characterization, and clinical testing. AI-driven analytics can identify patterns linking polymer chemistry, process parameters, and CQAs such as particle size, stability, and release kinetics, thereby accelerating formulation optimization and reducing experimental workload.

The integration of AI-driven predictive tools within adapted QbD and SbD frameworks enables more anticipatory, risk-informed nanomedicine development. These approaches can predict the evolution of nanoscale attributes during manufacturing and biological interaction, strengthening links between quality, safety, and performance and supporting more transparent and consistent regulatory evaluation.

Coupled with digital twins and physiologically based pharmacokinetic (PBPK) models (Khakpour et al., 2025), predictive simulations can forecast *in vivo* performance, biodistribution, and toxicity profiles with increasing accuracy, helping to refine designs before clinical testing. At the same time, global regulatory harmonization (Caputo et al., 2024), through coordinated efforts among different regulatory authorities worldwide, is essential to ensure these advances lead into equitable patient access. Divergent requirements for nanomedicine classification, characterization, and quality assessment currently create significant barriers to international development and approval (Rodríguez-Gómez et al., 2025). Establishing aligned guidelines for data standards, AI validation, and model-informed regulatory submissions would streamline approval pathways, foster transparency, and enable cross-border manufacturing and distribution of safe, high-quality PN therapeutics.

Together, AI-driven innovation and regulatory convergence will be central to realizing the full clinical and societal potential of PNs.

## 7 Conclusion

PNs represent one of the most dynamic frontiers in pharmaceutical innovation, yet their clinical progression remains constrained by analytical, manufacturing, and regulatory challenges. Despite major advances in polymer chemistry and nanocarrier design, regulatory evaluation still relies largely on conventional approaches that do not adequately capture nanoscale complexity, resulting in case-by-case decision-making and limited predictability. Addressing this gap requires international standards that integrate physicochemical characterization, biological evaluation, and QbD principles into coherent, reproducible methodologies. Future progress will depend on the convergence of SbD approaches, real-time process analytics, and emerging digital technologies such as AI and modeling, which can enhance predictability, optimize production, and strengthen regulatory confidence. At the same time, regulatory agencies must continue fostering open dialogue with academia and industry to accelerate the safe, transparent, and evidence-based approval of PNs. Ultimately, achieving global harmonization and methodological rigor will transform PNs from promising laboratory constructs into clinically reliable, patient-centered therapeutics capable of addressing complex and unmet medical needs.
